# Progress in tear microdesiccate analysis by combining various transmitted-light microscope techniques

**DOI:** 10.1186/s40659-016-0089-0

**Published:** 2016-06-03

**Authors:** Felipe Traipe-Salas, Leonidas Traipe-Castro, Daniela Salinas-Toro, Daniela López, Felipe Valenzuela, Christian Cartes, Héctor Toledo-Araya, Claudio Pérez, Remigio López Solís

**Affiliations:** Cellular and Molecular Biology Program, Faculty of Medicine-ICBM, University of Chile, Independencia 1027, Independencia, Postal code 8380453 Santiago, Chile; Fundación Oftalmológica Los Andes Ophthalmology Clinic (FOLA), Las Hualtatas 5951, Vitacura, Postal code 7650710 Santiago, Chile

**Keywords:** Tear, Tear ferning test, Transmitted-light microscopy, Tear microdesiccate, Dry eye

## Abstract

**Background:**

Tear desiccation on a glass surface followed by transmitted-light microscopy has served as diagnostic test for dry eye. Four distinctive morphological domains (zones I, II, III and transition band) have been recently recognized in tear microdesiccates. Physicochemical dissimilarities among those domains hamper comprehensive microscopic examination of tear microdesiccates. Optimal observation conditions of entire tear microdesiccates are now investigated. One-μl aliquots of tear collected from individual healthy eyes were dried at ambient conditions on microscope slides. Tear microdesiccates were examined by combining low-magnification objective lenses with transmitted-light microscopy (brightfield, phase contrasts Ph1,2,3 and darkfield).

**Results:**

Fern-like structures (zones II and III) were visible with all illumination methods excepting brightfield. Zone I was the microdesiccate domain displaying the most noticeable illumination-dependent variations, namely transparent band delimited by an outer rim (Ph1, Ph2), homogeneous compactly built structure (brightfield) or invisible domain (darkfield, Ph3). Intermediate positions of the condenser (BF/Ph1, Ph1/Ph2) showed a structured roughly cylindrical zone I. The transition band also varied from invisibility (brightfield) to a well-defined domain comprising interwoven filamentous elements (phase contrasts, darkfield).

**Conclusions:**

Imaging of entire tear microdesiccates by transmitted-light microscopy depends upon illumination. A more comprehensive description of tear microdesiccates can be achieved by combining illumination methods.

## Background

Desiccation of microvolumes of tear fluid on a flat glass surface at ambient conditions followed by a morphological assessment by light microscopy of the non aqueous remains has been widely used as a laboratory test in the assessment of patients suspected of Dry eye disease [1–6]. To date, such characterization has almost exclusively consisted in the observation of the occurrence of fern-like crystalloids [6–9]. Such method gained popularity after Rolando included a four-level scoring scale, from I to IV, whereby score I stands for abundant fern-like crystalloids (healthy tears) and, at the other end, score IV stands for the absence of those fern-like crystalloids (altered tears) [10, 11]. The method is commonly named as tear ferning test [1]. Recent studies have shown that tear microdesiccates are much more complex structures which are regularly formed by four main discrete concentrically organized morphological domains or zones. Fern-like crystalloids are just a part of such complexity [12]. A first domain (zone I), which is the one of earliest formation during desiccation, is formed by a hyaline material that surrounds the whole area of the tear microdesiccate and exhibits a variable number of transverse and highly refringent structures that resemble fractures or cracks. A second domain or zone II comprises a band of very homogeneous fern-like or leaf-like crystalloids emerging centripetally from regularly spaced points in proximity to zone I. A third domain (zone III) corresponds to the centermost area of the desiccate and is characterized by the presence of major crystalloid structures differing in robustness, length and branching. Finally, the transition band, is a morphologically distinct domain with the appearance of a narrow strip which is located along the entire interphase between zones I and II and whose relevance seems to be associated with the organization of the major morphological domains I and II [12, 13]. All those morphological domains have been jointly described on the basis of single observations by transmitted-light microscopy, particularly the one corresponding to the dark-field variant [12, 13]. Although such procedure is focused on the analysis of the whole tear microdesiccate, it probably misses objective and relevant data from particular domains of tear desiccates because it is based on a single setting of the light microscope. Such omission would be particularly relevant considering that the whole set of features of tear microdesiccates can be a direct reflection of the complex tear composition. In this regard, the assessment of tear desiccates should consider the examination of all four morphological domains instead of the sole consideration of fern-like crystalloids as it usually happens [6–9]. In the present study microscope settings were adjusted for the assessment of any single tear microdesiccate so that each of the main microdesiccate domains could be observed under optimized experimental conditions. In addition to confirm the occurrence of the four main morphological and structural domains in normal tear microdesiccates novel insights into the organization of some of these tear specimens have been gained.

## Results

### Volume of tear fluid for viewing entire microdesiccates under the microscope

**Fig. 1:**
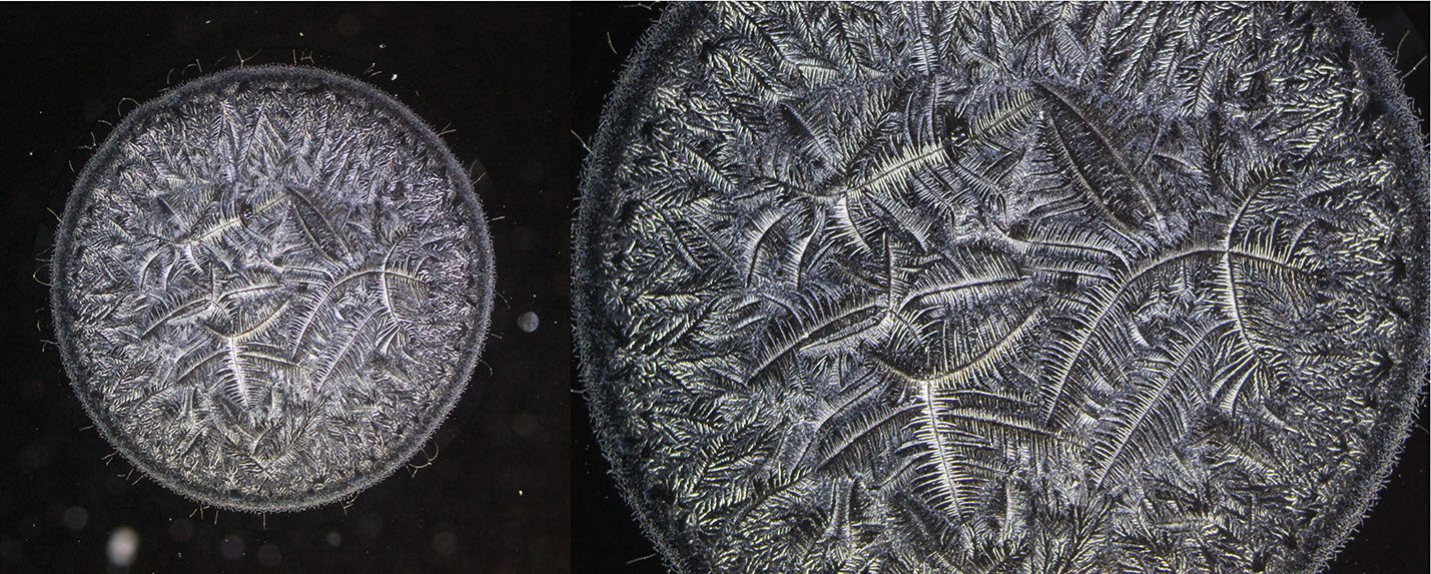
Image capture of entire tear microdesiccates by using low power objective lens. Digital image of an entire microdesiccate (about 3 mm diameter) produced from a 1 µL-aliquot of tear was captured by using a microscope fitted with 10× eyepieces and a 2.5× objective lens (*left*). Image capture was only partial when the objective lens was replaced by a conventional 5× objective lens (*right*)

**Fig. 2:**
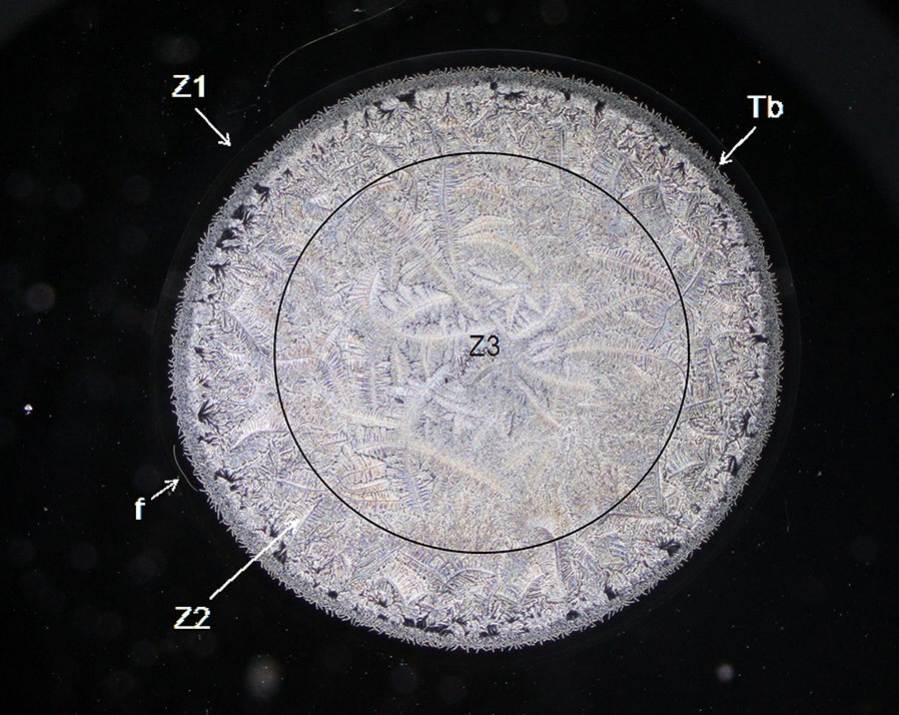
Morphological zones in a normal tear microdesiccate imaged with phase contrast (Ph3) microscopy. A hardly seen hyaline zone I *Z1* surrounds the whole circular area of the body of the tear desiccate in close proximity to a clearly structured transition band *Tb*. At the centermost area of the desiccate (demarcated by a *black* circumference) abundant major fern-like crystalloids feature zone III *Z3*. A compact homogeneous band of short fern-shaped or* leaf-shaped* crystalloid structures located between the transition band and zone III represents zone II *Z2*. Some bright filamentous structures (f) can be also seen as part of zone I

Conventional tear microdesiccates are produced when aliquots of 1–3 μL of tear fluid taken from a single eye are allowed to dry on a horizontal glass surface [12]. Volumes of tear fluid over 1.5 μL consistently generate microdesiccates covering a surface usually higher than the largest observation fields of standard light microscopes fitted with a 4–5× objective lens. Thus, for comparison purposes the present study was focused on microdesiccates produced from 1 μl of tear fluid whose circular images (about 3 mm diameter) were captured by using microscopes fitted with 10× eyepieces and a 2.5× objective lens (field of view 10.62 mm) (Fig. 1). Under current experimental conditions in this study, desiccation of those tear aliquots usually took place in about 7–8 min and tear microdesiccates were highly reproducible [13]. In effect, multiple tear microdesiccates produced from identical aliquots taken from a single sample of tear fluid showed marked similarities in terms of morphological features and distribution of their main morphological domains (zones I through III and transition band) (Fig. 2). By contrast, tear microdesiccates produced simultaneously from identical aliquots of tear fluid sampled from different healthy subjects usually presented marked differences from each other respecting morphological and structural features although they could exhibit a common design based on the occurrence of domains I through III and a transition band (see below).

### Microscopic observation of single tear microdesiccates using different transmitted-light techniques

**Fig. 3:**
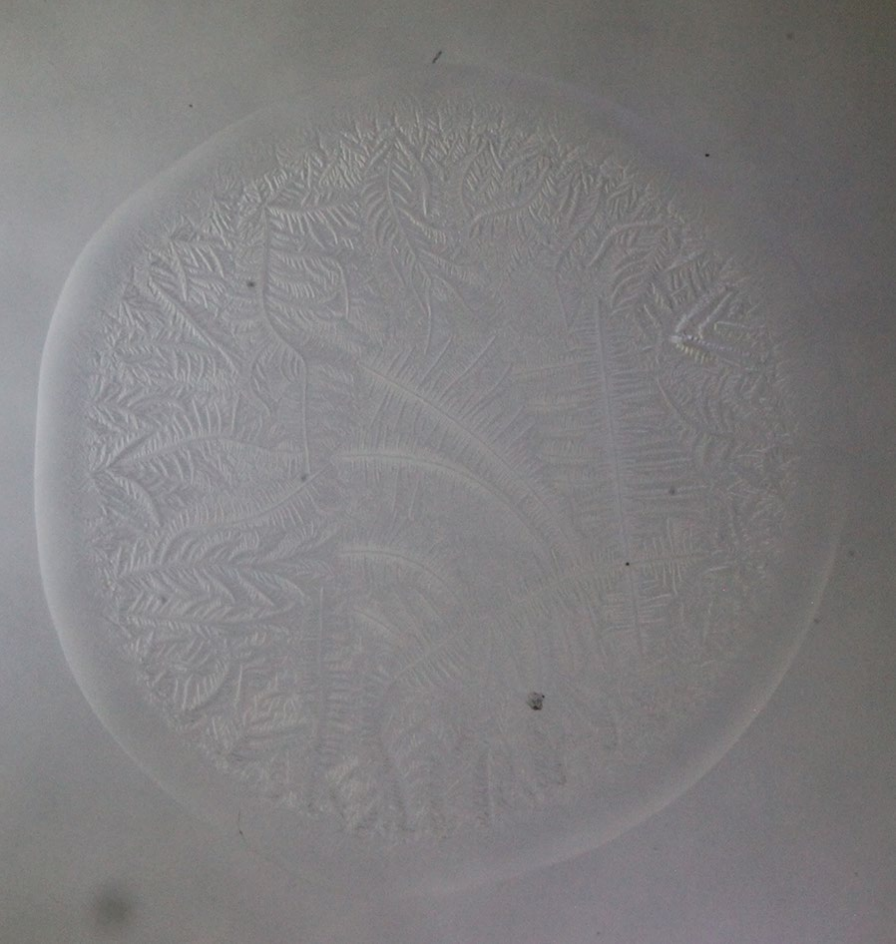
Representative image of a normal tear microdesiccate as observed by transmitted-light microscopy with bright-field illumination. A tear microdesiccate was produced from 1 µL of a tear sample and then observed with a microscope fitted with a 10× eyepiece and a 2.5× objective lens and a universal 5-position condenser system turret. Zone I is seen as a homogeneous bulky continuous structure that surrounds a mass of highly diverse poorly defined crystalloids

**Fig. 4:**
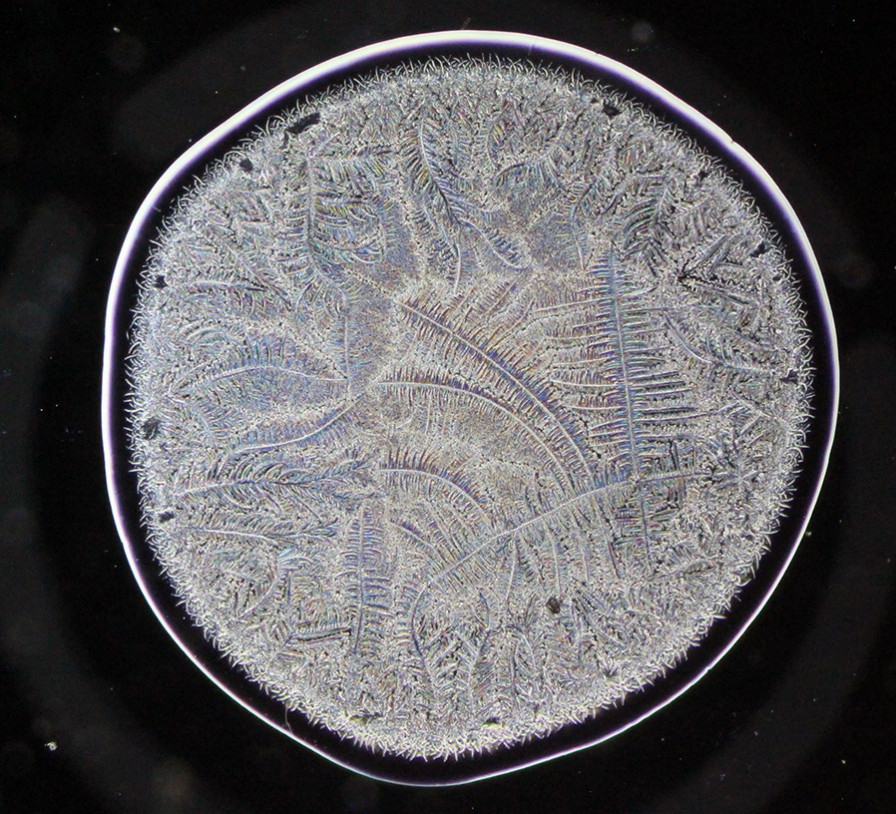
Representative image of a normal tear microdesiccate as observed by transmitted-light microscopy with phase 1 illumination. Both tear microdesiccate production and the lens system of the microscope were those described in the legend to Fig. 3. The bright halo of light on the border of the microdesiccate defines a thick morphological zone I surrounding a complex mass of tear crystalloids

**Fig. 5:**
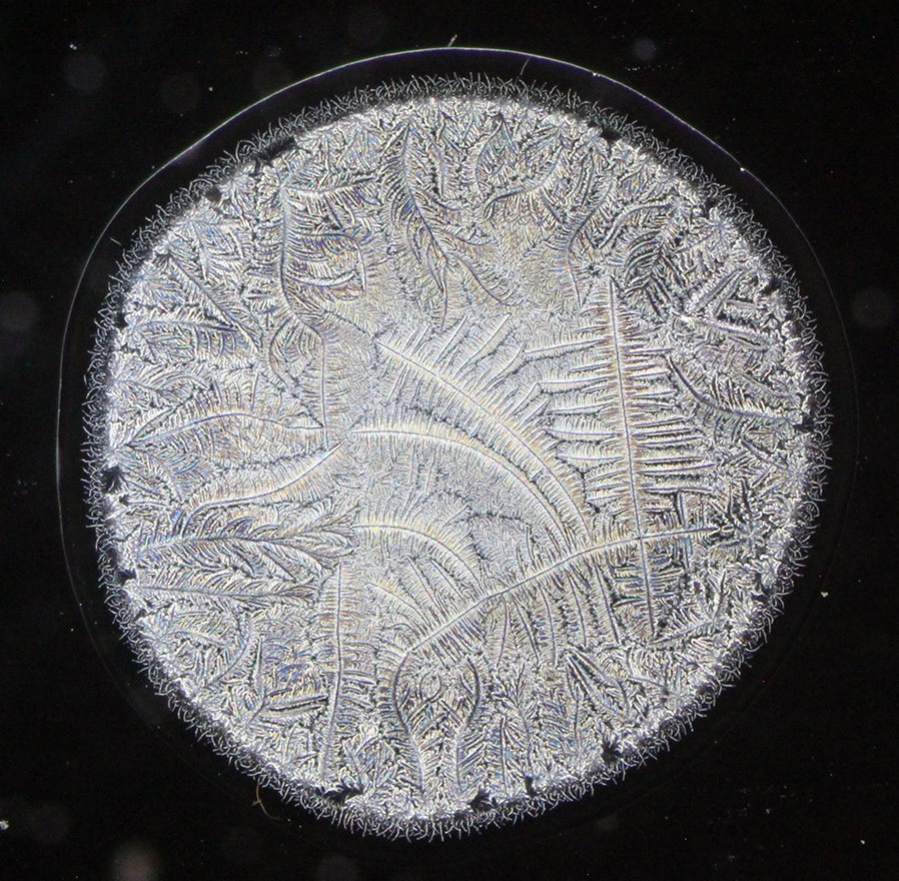
Representative image of a normal tear microdesiccate as observed by transmitted-light microscopy with phase 2 illumination. Both tear microdesiccate production and the lens system of the microscope were those described in the legend to Fig. 3. Both the outer border of zone I and its close contact with a well-defined transition band are two main features of tear microdesiccates derived from Ph2 illumination. Fern-like crystalloids of zones II and III are also seen

**Fig. 6:**
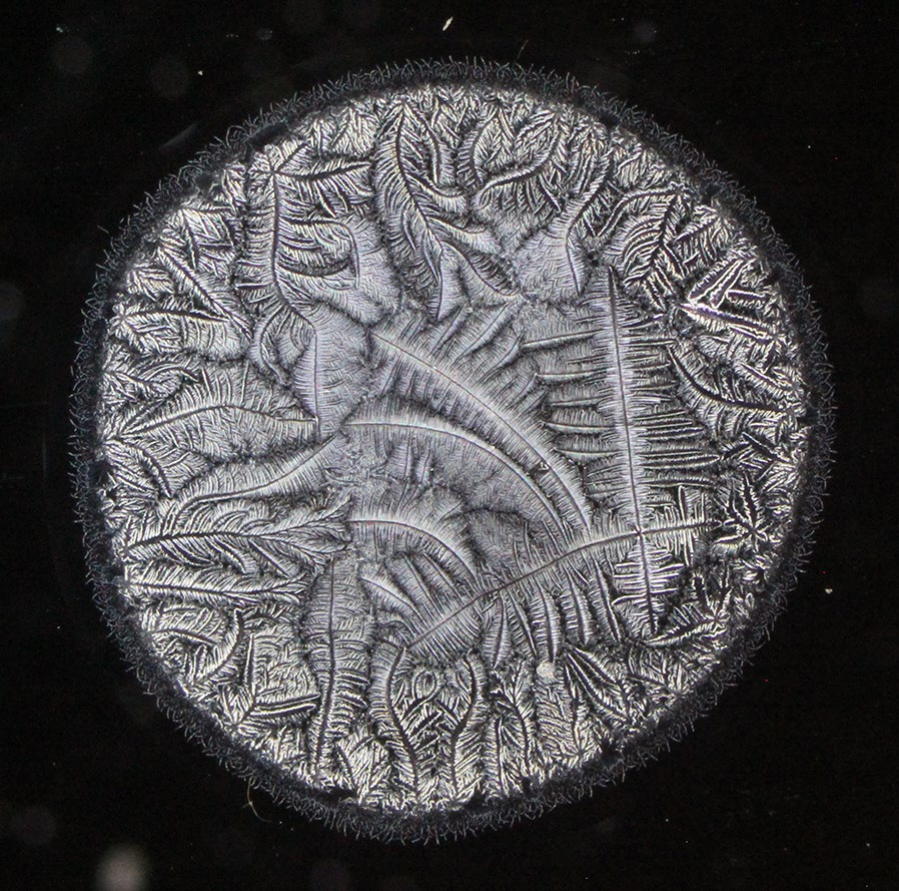
Representative image of a normal tear microdesiccate as observed by transmitted-light microscopy with phase 3 illumination. Both tear microdesiccate production and the lens system of the microscope were those described in the legend to Fig. 3. Zone I is not visible but the transition band is well-defined. In addition, optimum contrast of fern-like crystalloids is achieved so that the border between zones II and III can readily be identified

**Fig. 7:**
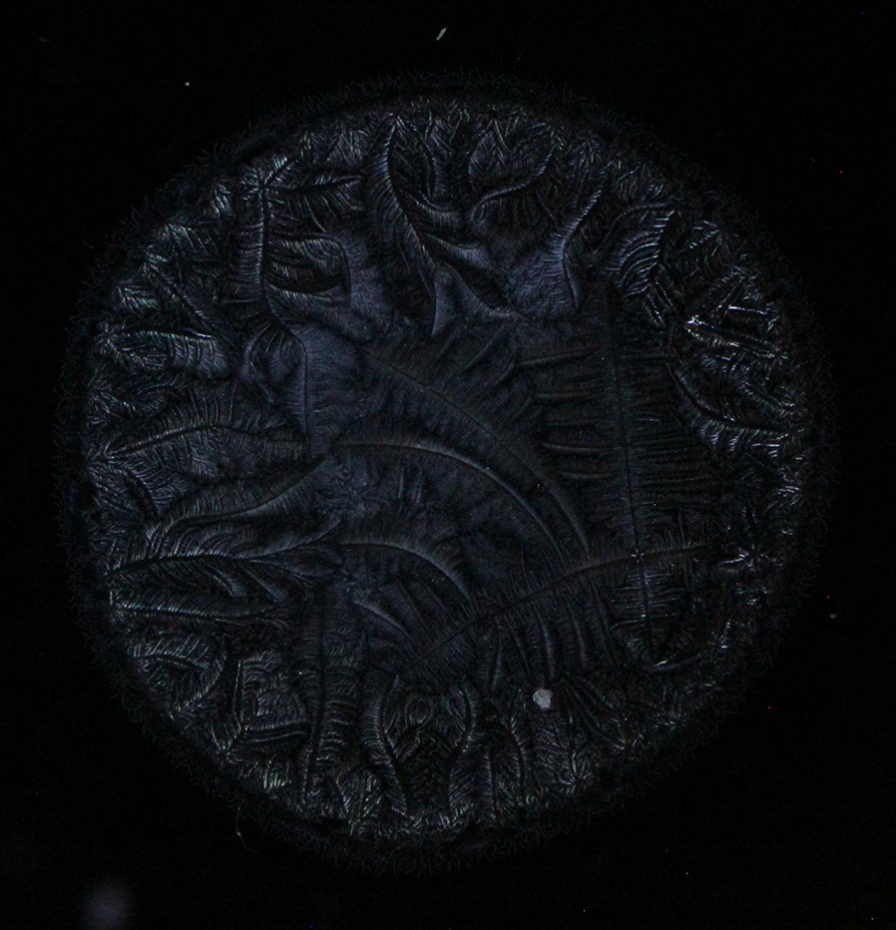
Representative image of a normal tear microdesiccate as observed by transmitted-light microscopy with dark-field illumination. Both tear microdesiccate production and the lens system of the microscope were those described in the legend to Fig. 3 Zone I is not visible, the transition band becomes somewhat diffuse and fern-like crystalloids become the main visible structures as a consequence of light-scattering

Every single tear microdesiccate was observed through an orderly sequence of transmitted-light brightfield (BF), phase contrast (Ph1, Ph2, Ph3) and darkfield (DF) microscope techniques. As shown in Figs. 3, 4, 5, 6 and 7, each of those techniques provided different but complementary images. Transmitted-light brightfield microscopy of tear desiccates provided sufficiently contrasted images showing a regular mostly homogeneous bulky continuous structure (zone I) serving as an external boundary for the whole microdesiccate (Fig. 3). Such feature was better defined by lowering of light intensity. Toward the inside of the desiccate and close to zone I, poorly defined bunches of relatively small fern-like structures could be seen. These would represent zone II. A few major fern-like crystalloid structures in zone III at the center of the microdesiccate were hardly seen. The transition band was not seen at all. Transmitted-light phase contrast technique for observing tear microdesiccates was conducted using successively the phase stop positions Ph1, Ph2 and Ph3 of a universal 5-position condenser system turret. Using the stop position Ph1, a translucent perimetral band (zone I) was a remarkable feature of microdesiccates (Fig. 4). With this observation method, the external border of zone I represented a well-defined limit of the whole microdesiccate. Individual or interconnected filamentous structures crossing the whole width of zone I were visible. This was a highly variable feature among microdesiccates from different subjects. The internal border of zone I was now shown to be in contact with a distinctive structured transition band. Using Ph1, the transition band could be seen as composed of highly interwoven filamentous elements (invisible with the brightfield microscopy!) delimiting the whole mass of tear crystalloids. Ph1 microscopy also showed that fern-like and leaf-like centripetally oriented structures emerge from the transition band and seem to be anchored to it. Though individually those structures were not well-defined, as a group they configured a better defined zone II of tear microdesiccates. Likewise, the centermost part of the desiccate (zone III) could be identified by default after the identification of the inner limits of zone II but own structures of zone III could be seen without much structural details. When the stop position of the condenser was changed to Ph2, zone I became barely visible although some filamentous structures crossing it (whose number was dependent upon the particular tear sample) were easily seen (Fig. 5). By this observation method a well-defined transition band consisting of highly convoluted filamentous components with nitid limits both toward zone I and zone II could be appreciated. Toward the center of the microdesiccate, crystalloids of zones II and III became better resolved. Using the stop position Ph3 of the condenser, zone I fully disappeared and filamentous elements previously seen as part of this zone were readily visible without a wrapping structure (Fig. 6). The transition band could be seen as an even more discrete zone of the microdesiccate displaying a clear distinction from zone II. Toward the center of the microdesiccate, Ph3 allowed to remark the minor and major crystalloids from zones II and III, respectively, and to set clear-cut differences between those zones. Fine structural details of those crystalloids, as well as their differential distribution in the microdesiccate, could be readily appreciated. Finally, using the transmitted-light darkfield technique and with a proper control of the intensity of light coming out of the condenser, zone I was mostly invisible but the presence of (a variable number of) transverse filamentous structures revealed its presence (Fig. 7). Although less evident, the transition band was again the outermost structured visible component of the desiccate. In addition, crystalloids of zones II and III became properly resolved. With this observation technique, both main and secondary axes of major crystalloids occurring in zone II, and a fraction of those occurring in zone III, displayed strong light-scattering properties. A summary of the effects of illumination on the main features of tear microdesiccates is shown in Table 1.

**Table 1:** Summary for the influence of illumination on the visibility of the main features of tear microdesiccates

	BF	Ph1	Ph2	Ph3	DF
Zone I	++	++	+	–	–
Zone I filaments	–	+	++	++	+
Bulky appearance of zone I	++	+	–	–	–
Transition band	–	++	+++	+++	+
Zone II-ferns	+	+	++	+++	++
Zone III-ferns	+	++	++	+++	++

*BF* brightfield; *Ph1* Phase 1 contrast; *Ph2* Phase 2 contrast; *Ph3* Phase 3 contrast; *DF* darkfield

Visibility:– nil; + scarce; ++ intermediate; +++ high

### Optimizing the observation of entire tear microdesiccates by transmitted-light microscopy

**Fig. 8:**
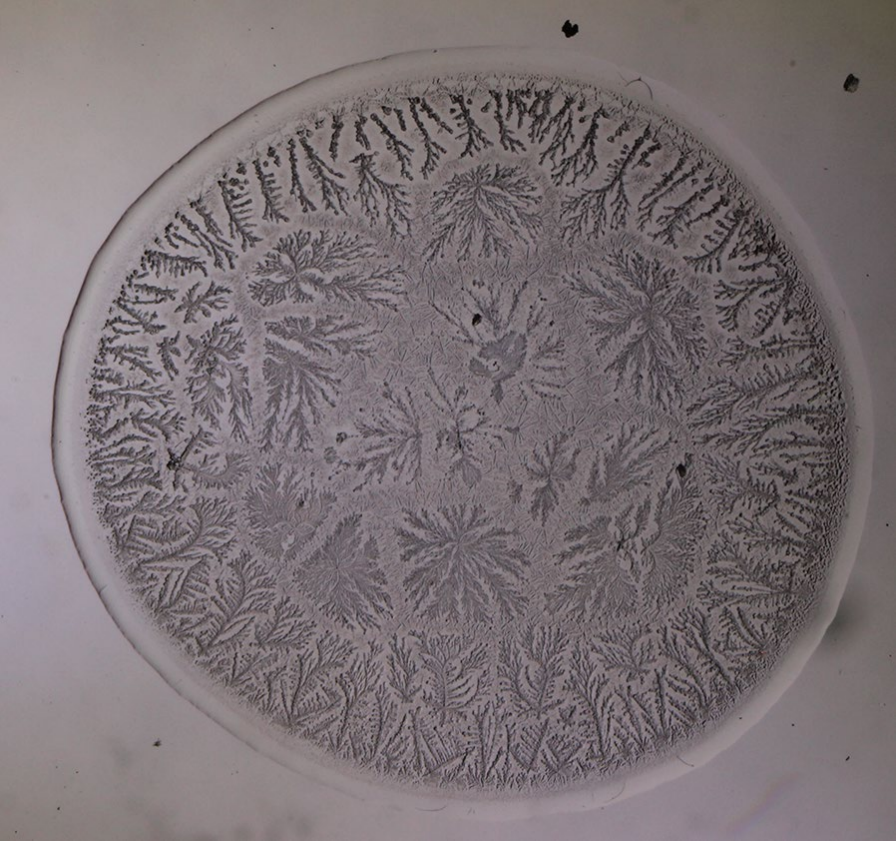
Representative image of a normal tear microdesiccate as observed by transmitted-light microscopy and BF/Ph1 illumination. Both tear microdesiccate production and the lens system of the microscope were those described in the legend to Fig. 3. Both a roughly cylindrical continuous zone I, a complex transition band and the structured body of the tear desiccate displaying a variety of major and minor crystalloids could be jointly appreciated. Some filaments can be seen as integral parts of zone I

**Fig. 9:**
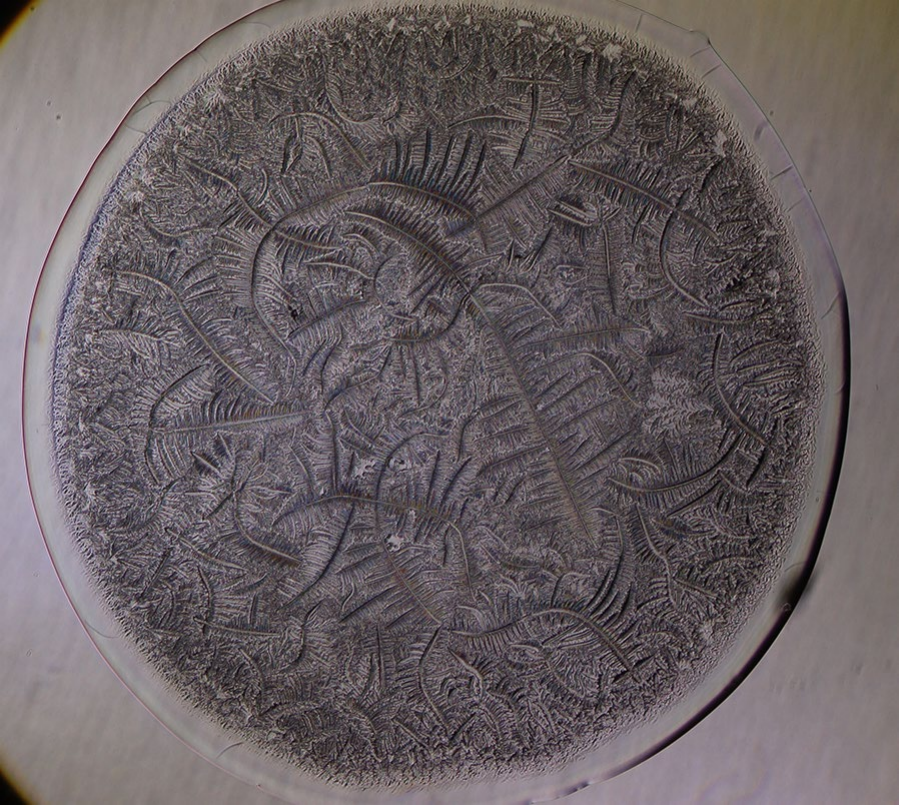
Representative image of a normal tear microdesiccate as observed by transmitted-light microscopy with Ph1^+^/Ph2 illumination. Both tear microdesiccate production and the lens system of the microscope were those described in the legend to Fig. 3. Again, both a roughly cylindrical continuous zone I, a complex transition band and the structured body of the tear desiccate displaying clearly defined major and minor crystalloids could be jointly appreciated. Filaments can be also seen as integral parts of zone I

**Fig. 10:**
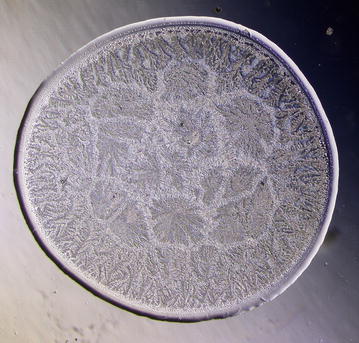
Representative image of a normal tear microdesiccate as observed by transmitted-light microscopy with Ph1/Ph2^+^ illumination. Both tear microdesiccate production and the lens system of the microscope were those described in the legend to Fig. 3. Also, both a roughly cylindrical and well-defined continuous zone I, a complex transition band and the structured body of the tear desiccate displaying major and minor crystalloids could be jointly appreciated. Filaments in zone 1 look like well-defined carvings

**Fig. 11:**
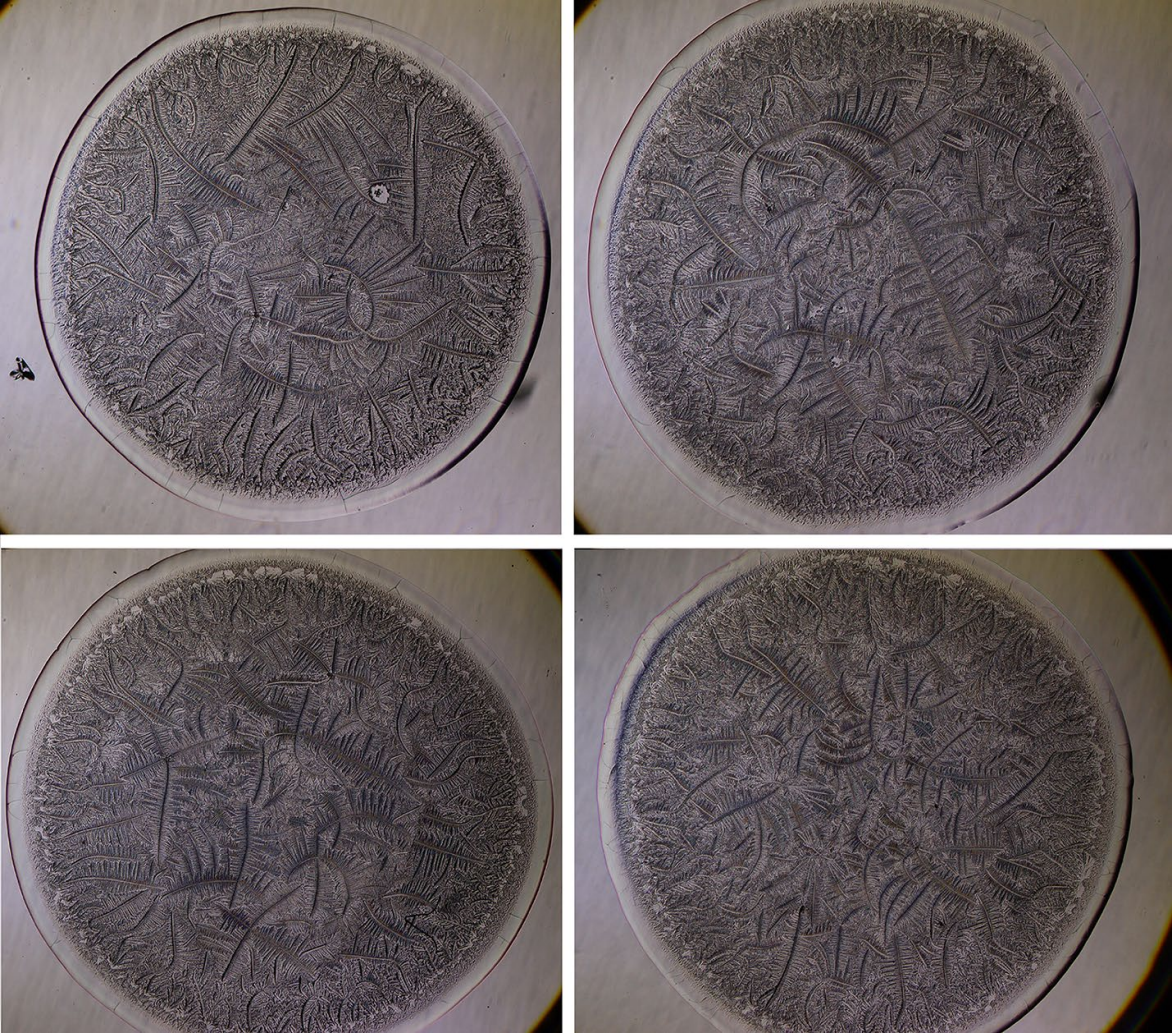
Reproducibility of tear microdesiccates produced from single healthy subjects and imaged with Ph1^+^/Ph2 microscopy. Microdesiccates produced from quadruplicate 1-µL aliquots of a single sample of tear taken from a healthy subject and observed by transmitted-light microscopy with Ph1^+^/Ph2 illumination displayed marked similarities concerning features of the four main morphological domains. Digital images of microdesiccates were captured at 25× magnification (10× eyepiece and 2.5× objective lens)

Apart from data collection from tear microdesiccates using the standard fixed positions of a universal 5-position condenser system turret, additional observations were conducted by setting the condenser in intermediate positions between some of the standard ones. This study aimed at getting single images of tear desiccates showing optimally their four main morphological domains. Because post-Ph2 positions in the turret disk, including Ph3 and DF, had resulted in a marked invisibilization of zone I, the new observations were focused on positions between BF and Ph1 as well as between Ph1 and Ph2. Turret positions between BF and Ph1 (Fig. 8) as well as others between Ph1 and Ph2 (Figs. 9, 10) produced microdesiccate images in which both zone I and the structured body of the tear desiccate could be jointly appreciated. Using these settings, zone I could be seen as a continuous seemingly compact cylindrical structure that borders the whole desiccate (Figs. 8, 9, 10). A highly variable number of filamentous substructures appeared as integral parts of zone I. Thus, condenser positions over the range Ph1/Ph2 but closer to Ph2 (Ph1/Ph2^+^) showed the filamentous substructures as carvings on zone I (Fig. 10). On the other hand, the transition band could be seen as a complex substructure displaying morphological differences when observed by setting the condenser at various positions over the intermediate ranges BF/Ph1 and Ph1/Ph2. In addition, the body of the desiccate displayed a variety of major and minor crystalloids, including fern-like structures. Altogether, positions over the range Ph1–Ph2, either closer to Ph1 (Ph1^+^/Ph2) (Fig. 9) or closer to Ph2 (Ph1/Ph2^+^) (Fig. 10) proved successful in producing “balanced” images of desiccates in which the main features of both zone I, transition band, zone II and zone III could be jointly observed. Such images were highly reproducible, that is, microdesiccates produced out of several tear samples taken from single healthy subjects and analyzed using selected illumination conditions over the range Ph1/Ph2 showed with no exception highly similar morphological profiles (Fig. 11).

## Discussion

In this study we have identified experimental conditions that will support the assessment of tear microdesiccates by variants of light microscopy. To date, studies involving characterization of single tear microdesiccates have frequently used either darkfield microscopy or phase contrast microscopy and have been exclusively focused on assessing either presence or absence of fern-like crystalloids. Furthermore, consideration of any other structural element of microdesiccates being formed during tear water evaporation has been disregarded consistently [1, 8–11]. Recent studies using light microscopy have shown that a normal tear microdesiccate comprises several annularly distributed morphological domains or zones, namely zones II and III (two highly distinctive zones whose organization is based on fern-like crystalloids), a transition band (a narrow compact band whose structure is based on rope-like elements) and zone I (a translucent and barely visible outer circle of desiccates) [12]. By making adjustments in the method of focusing light onto the dry tear specimen by means of an Abbé condenser with 5-position turret (brightfield, phase contrasts 1, 2 and 3 and darkfield) we have now been successful in identifying optimal conditions for the differential (and simultaneous) observation of some of those structural domains of tear microdesiccates. Thus, by using illumination systems other than those of the usual darkfield or phase contrast microscopy, zone I became clearly noticeable as an architectural distinct component of normal tear microdesiccates. To date, zone I of tear microdesiccates has gone mostly unnoticed among tear fern analysts despite it was originally described in 1955 by Solé as an amorphous and barely visible structure which can be penetrated by tiny rod-like elements [14–16]. Furthermore, by means of finer adjustments consisting in positioning the condenser in intermediate positions between brightfield and phase 1, or between phases 1 and 2, both fern-like crystalloids and zone I, that is, the two most distinctive elements of a normal tear microdesiccate, could be seen simultaneously and with a properly balanced resolution.

An analysis of the specialized literature shows that practically none of the reports concerning tear microdesiccates—with the exception of that of Horwath et al. [17]—present either whole tear microdesiccates or their outer zone I [17–21]. Moreover, descriptions in those reports are referred only to presence or absence of tear fern-like crystalloids. Such observational bias of researchers and clinicians using light microscopy of tear microdesiccates as a diagnostic test for the assessment of the ocular surface seems to derive from very different sources. Firstly, production of tear microdesiccates on glass surfaces and their analysis by light microscopy is widely known as the tear ferning test. Certainly, such nomenclature draws attention to a single goal [10, 17, 18]. On the other hand, most of reports on tear microdesiccates do coincide in documenting just a very minor area of every single microdesiccate [2, 7, 8, 20, 21]. Considering both variety and abundance of structures and domains that are usually present in normal tear microdesiccates, such selection is by far a confounding factor. Another also important factor accounting for the only partial use of the information derived from any tear microdesiccate is the lack of standardization among the observation procedures used in different studies. Thus, observations reported to have been made at magnifications of either 10× [21–23], 40× [8], 25 and 125× [17], 40–100× [24], 100× [25], or even 400× [9], are hardly comparable. Moreover, reports rarely indicate the particular combination of ocular and objective lenses used in the observations, which can also preclude comparisons and be of great significance for appropriate data collection. Quite often, the areas of the fields of view corresponding to the above-mentioned range of magnifications do oblige authors to select a fraction of the tear microdesiccate to be exhibited as representative of the whole specimen. A quite closely related aspect leading to the same biased result can derive from the use of relatively large tear volumes to produce microdesiccates, so that it becomes impractical or impossible to watch the whole specimen. In our experience, microdesiccates produced with tear volumes equal or higher than 2 µL can hardly be seen under a common light microscope whose lowest power objective lens is usually around 4–5× [12]. Unfortunately, data on the volume of tear used to produce microdesiccates are rarely communicated in specialized reports. Also in reference to methodological aspects that may restrict markedly the information provided by a tear microdesiccate is the use of some particular types of light microscope techniques. Among studies dealing with characterization of tear microdesiccates, a majority involved the transmitted-light darkfield microscopy variant [12, 13, 17], while others used either phase contrast microscopy [24, 25], visible light microscopy [9, 10] or polarized-light microscopy [20]. Some reports do not provide sufficient technical data in this respect [15]. Morphological information obtained by using those different experimental approaches can differ markedly. Concerning tear microdesiccates we have now shown that darkfield microscopy enhances imaging of fern-like crystalloids but, in turn, makes zone I practically invisible.

In an already classical report aimed to systematize the assessment of tear microdesiccates, Rolando proposed the use of a 4-level numeric scale (I through IV) to evaluate the power of tear fluid to form fern-like crystalloids following spontaneous desiccation on a glass surface at ambient conditions [1, 10]. In addition, those authors showed that levels I and II (higher fern-forming capability) were more frequent among tear fluids collected from normal eyes whereas levels III and IV (lower fern-forming capability) were more common in tear fluids of patients with keratoconjunctivitis sicca [1, 10]. Given the remarkable diversity of procedures to produce and evaluate tear microdesiccates, it has not been surprising that the Rolando’s scale has been used or interpreted very differently by different authors (e.g. ref. [20] versus ref. [22]). Also, in a recent analytical study on typing tear microdesiccates in association with the tear ferning test, a new 5-point scale displaying improved discrimination, repeatability and reliability over the conventional Rolando’s scale was proposed in order to provide a better support to researchers and clinicians using the test [18]. Such study was also focused only on the fern-like crystalloids with no consideration to any other structural element of tear microdesiccates [18]. Despite these various technical, methodological and interpretive limitations, acceptable sensitivity and specificity values of the tear ferning test in screening Dry eye have been reported [20, 26, 27]. Certainly, the properties displayed by a tear microdesiccate should account at least partly for the quality of the tear fluid from which it is produced. In this context, the link made by Rolando between a morphological feature of tear microdesiccates and tear quality is highly valuable and should be given first consideration. In accordance with that premise, our study was aimed at defining basic experimental conditions allowing the observer to characterize whole tear microdesiccates being produced under standard conditions. Thus, the combined use of a tear volume of 1–1.5 µL to produce a microdesiccate and a 2.5× objective lens for its analysis represented primary conditions to recognize a whole microdesiccate. To resolve and characterize the main morphological domains of a tear microdesiccate the use of alternative illumination settings, in reference to the basic positions of a standard 5-position turret condenser, was found to be equally important. In this study, some of the domains of a tear microdesiccate could be consistently resolved by using particular types of illumination. In agreement with a number of previous reports, the major tear fern-like crystalloids can be properly resolved using darkfield microscopy or some types of phase contrast illumination (Ph3). However, under this type of illumination zone I of tear microdesiccated specimens becomes practically invisible. Contrarily, by using some phase contrast illuminations (Ph1) the borders of zone I become clearly demarcated but resolution of the centermost fern-like crystalloids is reduced significantly. On the other hand, because of the consistent lack of use of stains in the assessment of tear microdesiccates, brightfield microscopy has not been exploited yet for the assessment of tear microdesiccates. Accordingly, none of the standard positions of the 5-position condenser by itself has allowed to describe comprehensively a whole tear microdesiccate. In order to attain views of microdesiccates in which both the zone I and the domains displaying fern-like crystalloids were jointly resolved, additional illumination settings provided by intermediate positions between the five fix positions in the turret condenser were explored. Thus, illuminations of tear microdesiccates from healthy subjects provided by intermediate positions between the standard brightfield and phase 1 positions or between the standard phase 1 and phase 2 positions resulted in whole tear microdesiccates showing simultaneously both fern-like crystalloids of zones II and III, a compact and structured zone I and a complex transition band. Recently reported studies from our laboratory have shown that the main domains of tear microdesiccates have distinctive physicochemical characteristics [28]. In that regard, using energy dispersive X-ray analysis (EDXA) coupled to scanning electron microscopy, Pearce and Tomlinson showed the presence of sulphur (together with K+ and Cl−) at the edge of the dried teardrop but not in the fern-like crystalloids [29]. Thus, different domains of tear microdesiccates may contribute with particular structural or functional properties to the tear film covering the eye surface [30, 31]. In accordance with this postulate, both the occurrence of major crystalloids in zone III (a common feature among normal microdesiccates typed as Rolando’s scores I or II), together with a seemingly structured zone I (a novel feature shown in this study) can be viewed as structural elements of normal tear microdesiccates whose scrutiny may shed some light on tear quality. Altogether, the assessment of whole tear microdesiccates may become a highly valuable source of information on normality or abnormality of the tear fluid. Far from contradicting the Rolando’s link between an altered score in the tear ferning test and physiopathological abnormality of the tear fluid, our findings do complement, enrich and diversify the possibilities of linking advantageously the morphology of whole tear microdesiccates with structural, compositional and functional aspects of tear fluid in individual patients and eyes. Clinical research in that direction should shed important lights on this new consideration of tear microdesiccates.

## Conclusions

Imaging of entire tear microdesiccates by transmitted-light microscopy depends upon illumination. A more comprehensive description of tear microdesiccates on the basis of structural domains can be achieved by combining illumination methods (bright-field, phases 1–3 and dark-field). Optimal conditions for the differential observation of structural domains of tear microdesiccates were identified. Thus, zones II and III (fern-like crystalloids) and zone I (the outermost homogeneous continuous structure) can be considered now as the two most distinctive elements of a normal tear microdesiccate, Both of them can be seen simultaneously and with a properly balanced resolution by transmitted-light microscopy.

## Methods

### Subjects

Fourteen subjects (10 men and 4 women; age range 18–27 years old) served as healthy volunteers. All of them fulfilled the following criteria: (a) Ocular Surface Disease Index (OSDI) score of 12 or less [32], (b) Schirmer I score of 10 mm or more at 5 min [33], (e) Fluorescein break-up time (FBUT) score of 5 s or more [34], (f) Ferning score I or II [10, 11], (g) tear osmolarity (Tear Lab Osmolarity System^®^) of 316 m Osm/L or less [35]. In addition, all subjects were neither contact lens wearers nor artificial tear users and had not taken any medication during the 3 months before tear assessment. All subjects acted as unilateral tear donor volunteers and signed an Informed Consent. The study was conducted according to the recommendations of the Declaration of Helsinki and approved by both the Ethics Committee of the Faculty of Medicine, University of Chile and the Ethics Committee of Fondecyt (Fondo de Desarrollo Científico y Tecnológico)-Chile.

### Tear collection

From each eye a single 3-min tear sample was taken by using absorbing polyurethane mini sponges as detailed elsewhere [36]. Aliquots of each tear sample were taken for desiccation assays immediately after collection.

### Tear desiccation and image capture

Unless otherwise specified, from each fresh tear sample 1.0-μL aliquots were taken using a 2-μL Gilson micropipette fitted with a ultrafine tip and placed sharply on the center of individual glass microscope slides that had been positioned horizontally. Tear aliquots were allowed to dry spontaneously at ambient conditions (temperature range of 18–25 °C, relative humidity range of 36–40 % and 570 meters above sea level (MASL). Micrographs of the dry specimens, named microdesiccates, were taken using a Zeiss Axiostar Plus microscope (objective lens = 2.5×, ocular lenses = 10×) fitted with a universal 5-position condenser system turret (bright-field, Phases 1, 2 and 3 and dark-field) and with a Canon Powershot G10 14.7 megapixel digital camera. Microdesiccates were routinely prepared in triplicate and classified as types I through IV according to Rolando’s criteria [1, 10].

### Materials

Ultrafine micropipette tips (Natural round Microflex ™, round capillary section, OD 0.57 mm) were acquired from Therapak Pharma Services Ltd. (Hayes, Middlesex, United Kingdom). Glass microscope slides were obtained from W. Knittel Glass (Braunschweig, Germany). Mini-sponges for tear collection (Pele Tim, sizes 0 or 1) were purchased from VOCO GmbH (Cuxhaven, Germany).
